# Correction: Targeted lysosomal activation in bladder epithelium enhances clearance of intracellular uropathogenic *Escherichia coli*

**DOI:** 10.1371/journal.ppat.1014720

**Published:** 2026-10-23

**Authors:** Kathrin Tomasek, Kristina Skurvydaite, Gauri Paduthol, Allison M. Burns, Léa Schlunke, Valentin Borgeat, Christian Pasquali, Mario Romani, John D. McKinney

The third to sixth authors, Gauri Paduthol, Allison M. Burns, Léa Schlunke and Valentin Borgeat, should be noted as contributing equally to this work while the last three authors, Christian Pasquali, Mario Romani and John D. McKinne, should not have been attributed equal contribution to this work but as joint senior authors of this work.
