# Supplementary material for: Targeted lysosomal activation in bladder epithelium enhances clearance of intracellular uropathogenic Escherichia coli
Source: PLoS Pathog. 2026 Sep 25;22(9):e1014448. doi: 10.1371/journal.ppat.1014448 (PMC13614569; doi:10.1371/journal.ppat.1014448)
Supplement: Supplementary file 1 [file ppat.1014448.s015.pdf]

Source images for Fig 4E

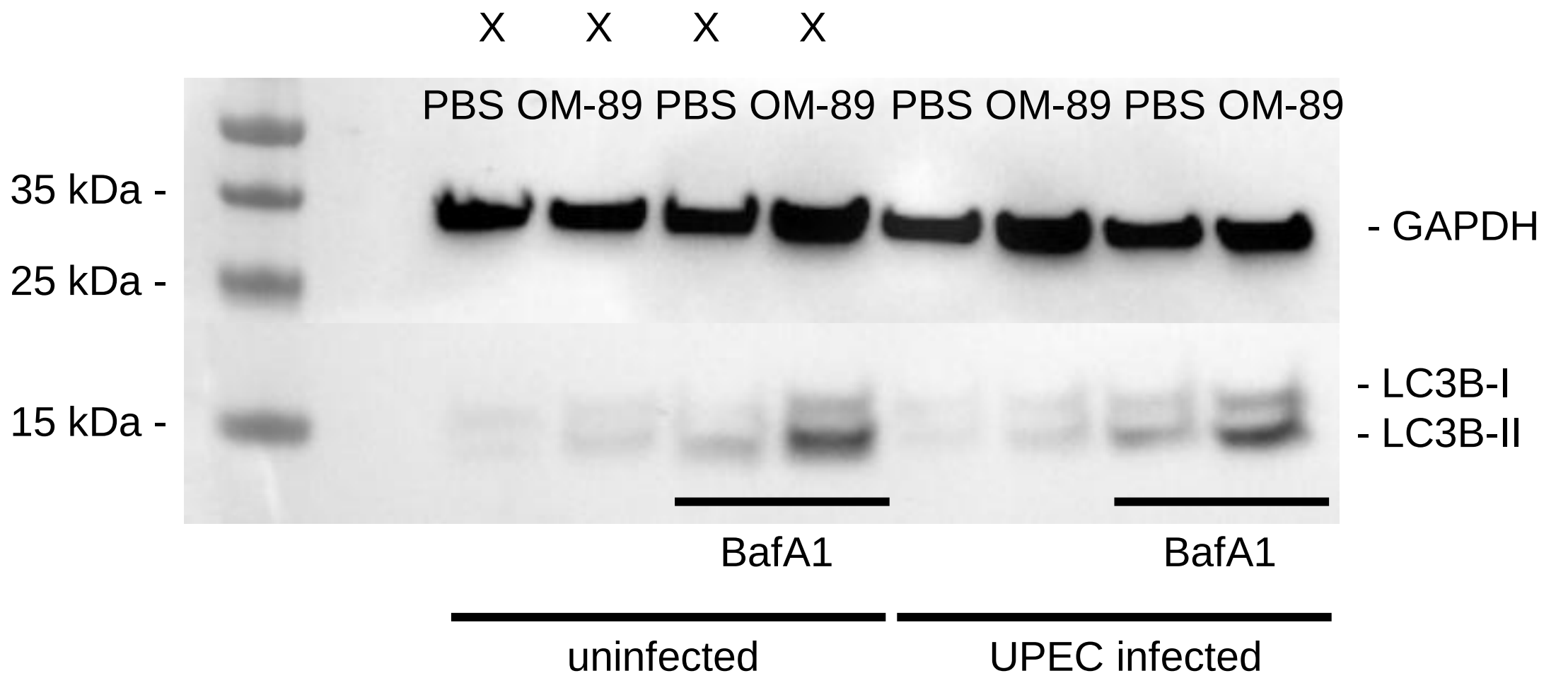

Source image for Fig 4E – autophagic flux in mouse bladder epithelial cells with OM-89 treatment. Lanes 1-8: PBS, OM-89, PBS+BafA1, OM-89+BafA1 (uninfected and UPEC infected, as indicated). Lanes marked "X" were not included in the published figure. Imaged on ChemiDoc XRS+ Molecular Imager from BIO RAD using colorimetric method to detect PageRuler Plus Prestained Protein Ladder 10-250 kDa from Thermo Scientific and chemiluminescence (HRP) for LC3B/GAPDH detection.

Source images for S7C Fig

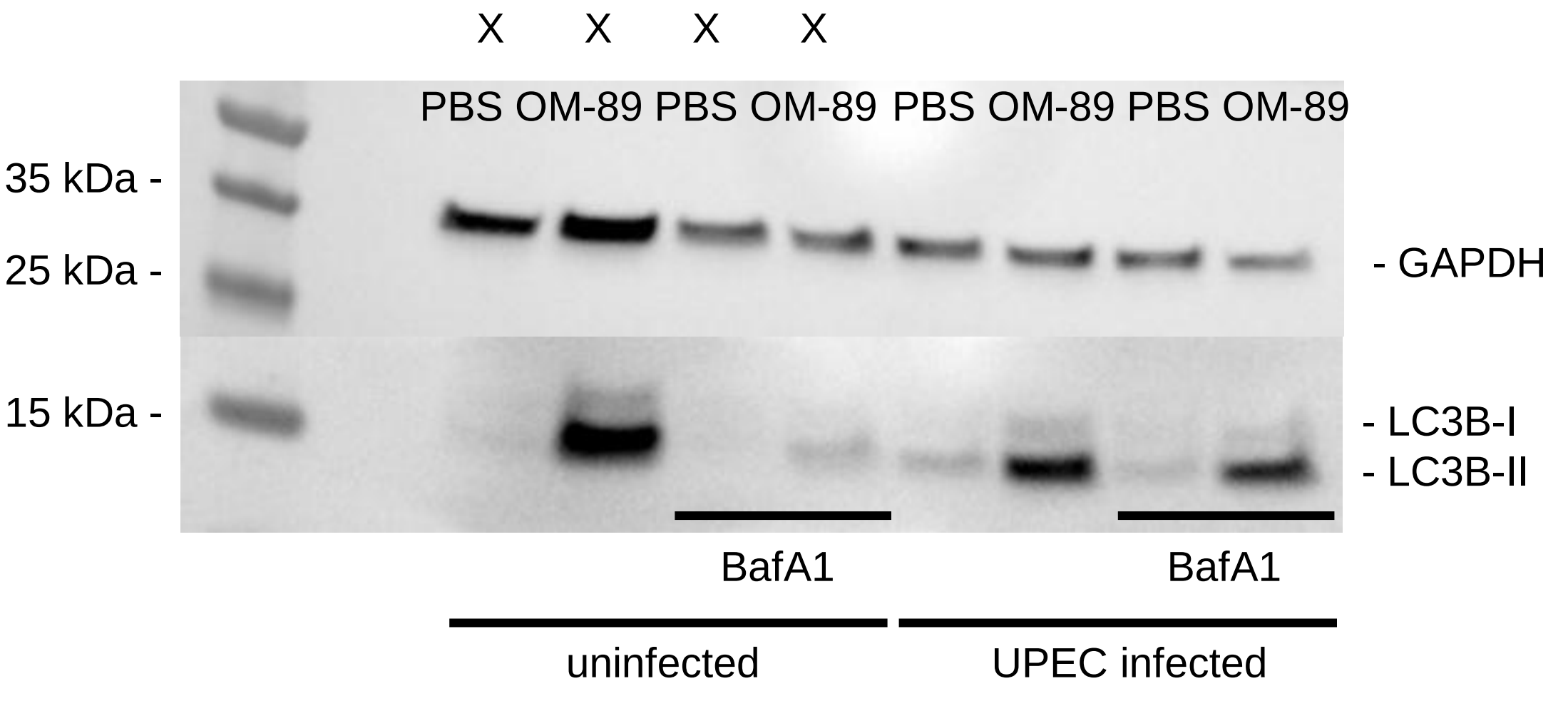

Source image for Fig 4E – autophagic flux in human bladder epithelial cells with OM-89 treatment. Lanes 1-8: PBS, OM-89, PBS+BafA1, OM-89+BafA1 (uninfected and UPEC infected, as indicated). Lanes marked "X" were not included in the published figure. Imaged on ChemiDoc XRS+ Molecular Imager from BIO RAD using colorimetric method to detect PageRuler Plus Prestained Protein Ladder 10-250 kDa from Thermo Scientific and chemiluminescence (HRP) for LC3B/GAPDH detection.
