# Supplementary material for: Targeted lysosomal activation in bladder epithelium enhances clearance of intracellular uropathogenic Escherichia coli
Source: PLoS Pathog. 2026 Sep 25;22(9):e1014448. doi: 10.1371/journal.ppat.1014448 (PMC13614569; doi:10.1371/journal.ppat.1014448)
Supplement: Supplementary file 2 [file ppat.1014448.s016.pdf]

Full Revision

Manuscript number: RC-2026-03491
Corresponding author(s): Kathrin, Tomasek

1. General Statements

We thank the reviewers for their constructive and thoughtful evaluations of our manuscript. Both reviewers considered the study technically rigorous and agreed that the conclusions are generally supported by the data, while identifying several opportunities to improve clarity, interpretation and presentation.

We are especially encouraged that both reviewers found the study to be of strong interest to the urinary tract infection (UTI) field, while recognizing the significance of identifying a previously uncharacterized mechanism of action for OM-89 (Uro-Vaxom®), a clinically established therapy for recurrent UTIs.

The reviewers primarily requested clarification of mechanistic interpretations, additional presentation of existing datasets and a more cautious framing of certain conclusions. We agree with these suggestions and have revised the manuscript accordingly. In particular, we refined the wording throughout the manuscript to avoid interpretations that extend beyond the data presented, clarified our conclusions, discussed the limitations of our study and adapted the manuscript title.

In response to the reviewers' comments, we further performed additional analyses of existing transcriptomic and imaging datasets and carried out complementary intracellular CFU experiments in differentiated bladder epithelial monolayers. These new data provide an orthogonal measure of intracellular bacterial burden and support the central conclusions of the study while adding further mechanistic insight into the epithelial effects of OM-89. Importantly, all newly generated data were consistent with, and further strengthened, the central conclusions of the study.

In the fully revised manuscript, we have updated Figure 1, Supplementary Figure 3, Supplementary Figure 4 and Supplementary Figure 7. Additionally, we have introduced a new Figure 2. To improve clarity and provide a more comprehensive presentation of the data within the main text, we transferred panel F from the previous Supplementary Figure 2 to replace panel I in the previous Figure 1. No data were removed during this process; rather, the original Figure 1I represented a subset of the data shown in Supplementary Figure 2F, and all data are now retained within the expanded panel. This panel is now presented as Figure 2A.

Overall, we are grateful for the reviewers' comments, which have substantially improved the clarity, rigor and translational positioning of the manuscript.

2. Point-by-point description of the revisions

Reviewer #1

Major points:

1. The data indicate that OM-89 exposure in the organoids enhances lysosomal degradation pathways and (in mBOs) autophagic flux, and the authors conclude this is a mechanism by which UPEC regrowth after antibiotic treatment (modeling rUTI) is inhibited by OM-89. They also show enhanced cellular uptake of fluorescently labeled antibiotics (ampicillin) in organoids - this leads them to conclude (and state in the paper's title) that increased intracellular antibiotic concentration effects increased killing of UPEC and decreased regrowth. These are two separate proposed mechanisms, and especially with regard to the antibiotics, they have not shown that increased intracellular antibiotic concentration actually kills intracellular UPEC in their model - only that regrowth as measured microscopically is less. In total, a mechanistic connection between the observed lysosomal effect and the intracellular antibiotic uptake, and which one is more important for UPEC control in this model, is incomplete. The precise wording of the paper's title should be reconsidered accordingly.

We agree with the reviewer that our study does not establish a direct mechanistic connection between OM-89-induced lysosomal remodeling and enhanced intracellular antibiotic accumulation, nor does it definitively determine the relative contribution of each process to intracellular UPEC control. Further studies dissecting the molecular pathways underlying these phenotypes will be required to determine whether they are mechanistically linked or represent parallel epithelial defense responses induced by OM-89.

Importantly, additional CFU experiments performed during revision (as suggested in point number 4) revealed that OM-89 already reduces intracellular bacterial burden following a classical gentamicin protection assay, prior to prolonged ampicillin exposure. These findings suggest that enhanced intracellular bacterial control cannot be explained solely by increased intracellular antibiotic accumulation and support a direct contribution of epithelial antimicrobial mechanisms, including lysosomal activation, to the observed phenotype. Nevertheless, the relative contribution of lysosomal remodeling and enhanced antibiotic uptake to bacterial clearance remains unresolved and will require further investigation.

Accordingly, we changed the title to "Targeted lysosomal activation in bladder epithelium enhances clearance of intracellular uropathogenic *Escherichia coli*." This revised title avoids implying a direct causal link between increased intracellular antibiotic accumulation and bacterial clearance while reflecting the central biological process identified in our study.

2. OM-89 is taken orally for rUTI prevention, and some "components" reach the urinary tract (line 81). But it isn't explained how applying OM-89 directly to organoids models how its components may reach the bladder epithelium (from the basolateral side, if the OM-89 is applied outside the

organoids) in the whole animal or human. At the least, this limitation should be stated in the Discussion.

We thank the reviewer for pointing out this limitation. Although advanced in vitro models help to better mimic the in vivo situation, they still do not fully recapitulate all aspects of drug exposure and delivery observed in vivo. We included the following statement of limitation now in the discussion in lines 493-503: "One limitation of our study is that OM-89 was applied directly to epithelial cultures and organoids, whereas in clinical use it is administered orally. Although pharmacokinetic studies have demonstrated systemic distribution and urinary accumulation of OM-89-derived components following oral administration (van Dijk, 1982), our experimental setup does not recapitulate the exact route, kinetics or concentration profiles encountered in vivo. Rather, our models were designed to determine whether bladder epithelial cells are capable of responding directly to OM-89-mediated signals and to identify the intracellular pathways involved. Given the documented systemic exposure following oral administration, direct effects on the urothelium are biologically plausible. However, future studies will be required to determine how the epithelial responses identified here integrate with the complex systemic and immune-mediated effects of OM-89 under physiological administration conditions."

3. In the lysosome studies starting on line 319, the cultured cells are all infected (and either treated with OM-89 or not). What observations regarding number and size of vesicles, etc (all the measures in Fig 6) are evident when cells are treated with OM-89 only? These data should be presented (at least as a supplemental figure) to enable optimal interpretation of the OM-89+UPEC data in Fig 6. As the authors themselves indicate, OM-89 may be having a generalized effect on endocytic and/or autophagic flux by bladder epithelial cells, independent of infection.

We thank the reviewer for this helpful suggestion and agree that assessing OM-89 treatment in the absence of infection provides important context for interpreting the infection-associated phenotypes as shown in Figure 6.

Accordingly, we have included additional supplementary data examining the effects of OM-89 alone in both murine and human bladder epithelial cells. Specifically, we added analyses of Lamp1-positive lysosomal vesicles, lysosomal acidification (LysoSensor), and Cathepsin L activity under uninfected conditions (Supplementary Figures 4A, 4G and 7D-F). We comment on these additional findings in the Result section in lines 242-246 and lines 366-370, and in the Discussion section in lines 469-483.

These experiments, together with the transcriptional data in SI Figure 3D, demonstrate that key features of lysosome-centered remodeling and activation are already induced by OM-89 in the absence of infection, indicating that OM-89 directly modulates epithelial lysosomal pathways rather than merely amplifying infection-driven responses. Inclusion of these data provides additional context for interpreting the infection-associated phenotypes shown in the main figures and further supports the concept of OM-89 as a direct modulator of epithelial antimicrobial function.

4. With the organoids, beyond the microscopic quantification of UPEC, can CFUs be measured?

We appreciate the reviewer's interest in obtaining orthogonal measurements of bacterial burden. Performing CFU quantification directly from microinjected organoids is technically challenging, as it requires highly reproducible injections into identical numbers of organoids while avoiding bacterial leakage into the surrounding extracellular matrix. Even minor variations or accidental release of bacteria into the Matrigel can substantially affect CFU recovery and compromise interpretation.

To address the reviewer's underlying question while avoiding these limitations, we performed intracellular CFU assays using differentiated mouse bladder epithelial monolayers. Following a classical gentamicin protection assay for 1 hour, OM-89-treated cells displayed significantly reduced intracellular bacterial burden compared with PBS controls (new Figure 2C). Addition of ampicillin for 3 hours after the gentamicin protection phase resulted in a similar trend but did not further significantly reduce the bacterial burden (new Figure 2D). We commented on these findings in the Results section in lines 169-182, and in the Discussion section in lines 463-469 and lines 474-483. We also updated the Methods section in lines 637-652 with the intracellular bacterial burden assay description.

These experiments provide an orthogonal readout of intracellular bacterial burden and are consistent with enhanced epithelial control of intracellular UPEC.

In addition, we would like to clarify that the higher-throughput microscopy approach used throughout the organoid experiments does not allow strict discrimination between luminal, intracellular and tissue-associated bacteria. We therefore revised the terminology throughout the manuscript and now consistently refer to the measured signal as "intra-organoid bacterial burden". To clarify this point, we added the following statement to the Results section (line 115): "Hence, the microscopy data represent the total "intra-organoid" bacterial burden at each experimental stage, without distinguishing the exact localization of the bacteria – which can be luminal, intracellular or tissue-associated.". Consistent with this clarification, we have replaced the term "antibiotic-mediated killing" throughout the manuscript with the more cautious wording "antibiotic-mediated clearance" or "reduced bacterial burden", where appropriate.

Minor points:

1. In Fig 1A, the "co-application" horizontal line is under the 7-10 hour window, but the text suggests that the application of antibiotics and OM-89 in this experiment is between 4-7 hours.

We thank the reviewer for pointing this out. Indeed, in the co-application regime, OM-89 is added at the same timepoint as the antibiotic – meaning straight after monitoring the growth phase at 4h post-infection (pi). We now adapted the horizontal line for the "co-application" treatment in Figure 1A accordingly to represent the time-point of OM-89 addition better. Additionally, we added a line for the antibiotic-treatment in order to further facilitate readability.

Review COMMONS

2. How are antibiotics and OM-89 "removed" at the 7-hour mark? This was not detailed in the Methods.

Although we had specified this in the methods section (now line 682: “For every media exchange (e.g. antibiotic treatment or withdrawal), each well was washed with 9 ml of the respective media before leaving 1 ml in the well.”), we realized the positioning was not optimal as we had mentioned this part under the point “Bacterial injection” in “Injection experiments”. We therefore now separated this part, together with the lid preparation, from the “Bacterial injection” part and created the new subsection “Lid preparation for media changes” (line 668 onwards).

3. What time point was used for the transcriptomic profiling of organoids? This is not clear from the relevant Methods or Results sections.

As stated in the methods section, RNA for transcriptomic profiling from mBOs was extracted at 4h post-infection (pi) (now line 892).

4. In showing that OM-89 "attenuated" the magnitude of inflammatory responses (Fig 2C and S3B), it would be helpful to add a panel showing the comparison of OM89+UPEC to PBS alone - this would be expected to convey activity (red) in the infection-related pathways, but to a lower magnitude than seen in UPEC vs PBS.

Please see our combined response at point 5.

5. Similarly, in the results outlined starting on line 196, it would be helpful to add a panel showing OM89+UPEC vs OM89 alone.

We thank the reviewer for these suggestions. We performed the requested additional analyses and generated Gene Ontology Biological Process (GOBP) enrichment plots comparing (i) PBS+UPEC versus PBS, (ii) OM-89+UPEC versus PBS and (iii) OM-89+UPEC versus OM-89.

As anticipated by the reviewer, these analyses show that infection-associated pathways remain induced in OM-89-treated infected organoids but with a reduced magnitude compared with infected PBS controls. Specifically, pathways that are strongly enriched in the PBS+UPEC versus PBS comparison display lower enrichment significance and effect size in the OM-89+UPEC versus PBS comparison. Furthermore, many of these pathways are no longer significantly enriched in the direct OM-89+UPEC versus OM-89 comparison, indicating that OM-89 attenuates the transcriptional inflammatory response induced by UPEC infection. These observations are consistent with our original interpretation, concluded from Figure 3C, that OM-89 dampens excessive infection-associated inflammatory signaling while preserving epithelial antimicrobial activity.

Importantly, we found that the direct comparison between PBS+UPEC and OM-89+UPEC, presented in the original Figure 3C, remains the most informative representation of the OM-89 effect because it controls for infection status while specifically highlighting the transcriptional changes induced by OM-89. By contrast, comparisons against PBS or OM-89 alone involve simultaneous changes in both infection and treatment status, making biological interpretation less straightforward.

Nevertheless, because the additional analyses directly address the reviewer's request and provide complementary context for interpreting Figure 3C, we have included them in Supplementary Figure 3B.

6. In line 236, what is meant by lysosomal "activation"? A more specific term should be chosen here.

We thank the reviewer for this question and aim to increase readability of this section. With lysosomal activation in the first sentence of the mentioned paragraph, we referred to the observed effect of upregulated lysosomal pathways and enhanced lysosomal function (measured by alterations in lysosomal vesicles) in the previous paragraph. However, to make the connection to the previous paragraph better, and given the comment number two of reviewer number two, we changed the whole first paragraph of this section. Therefore, the first sentence of this paragraph (line 252 onwards) reads now: “To test whether the observed effects on lysosomal pathways could mechanistically, at least in parts, explain OM-89-mediated protection, we first used Genebridge analysis (Li et al, 2019) to examine how the lysosomal gene signature identified in our RNA-seq data relates to host defense programs in the human bladder.”

7. In the Abstract (line 25), the phrase "Using bladder organoids..." is a dangling modifier.

We thank the reviewer for pointing this out and changed the sentence accordingly to “OM-89 promotes lysosomal acidification and increases lysosomal protease activity in bladder organoids and differentiated epithelial monolayers, thereby directing intracellular UPEC toward degradative compartments.” (now line 24)

Typographical and copyediting:

We thank the reviewer for identifying typographical errors and have corrected them throughout the manuscript.

2. Line 76 should read "when combined with antibiotic therapy..."

As this sentence is to emphasize the already observed protective effects of OM-89, and the two studies mentioned were either performed without or in combination with antibiotics, we changed the sentence to “For instance, rodent infection studies have demonstrated protective effects of OM-89 alone (Bosch et al, 1988; Lee et al, 2006) and in combination with antibiotic

therapy (Canton et al, 2025; Bessler et al, 2010), although this observed in vivo protection could not be linked to any major quantitative changes in bladder immune cell infiltration (Canton et al, 2025), leaving the underlying molecular mechanism not fully resolved." for better readability. (now line 71)

Reviewer #2

Major comments:

1. I think the authors overstate the novelty of the concept that the urothelium is an active targetable determinant of infection and treatment outcomes. This is not an entirely new concept since previous studies have examined antimicrobial peptides and other factors from the urothelium.

We thank the reviewer for this important point and agree that the urothelium has long been recognized as an active participant in host defense through mechanisms such as antimicrobial peptide production, pathogen sensing and regulation of inflammatory responses. We have therefore revised the manuscript to avoid implying that urothelial involvement in infection outcome is itself a novel concept. Instead, we now emphasize the specific advance of our study: the identification of lysosome-centered epithelial activation as a therapeutically targetable mechanism that enhances intracellular bacterial clearance and potentiates antibiotic efficacy.

In the abstract we changed: "Our findings position the bladder epithelium from a passive barrier to an active, targetable determinant of treatment outcome and suggest host-directed modulation of epithelial antimicrobial pathways as a promising strategy to enhance intracellular bacterial clearance." to "Our findings demonstrate that bladder epithelial antimicrobial pathways can be pharmacologically reinforced to influence treatment outcomes by enhancing intracellular bacterial clearance." in line 29.

In the introduction we changed: "Together with increased intracellular accumulation of antibiotics across different classes, this leads to improved intracellular killing and reduced bacterial regrowth across diverse UPEC strains." to "Together with increased intracellular accumulation of antibiotics across different classes, these changes are associated with improved intracellular clearance and reduced bacterial regrowth across diverse UPEC strains." in line 90 and "Together, these findings reveal a previously unrecognized epithelial lysosome-centered mechanism by which OM-89 enhances intracellular antibiotic performance and repositions the bladder epithelium from a passive reservoir of infection reactivation to an actively transformable antimicrobial compartment influencing treatment outcomes." to "Together, these findings reveal a previously unrecognized lysosome-centered epithelial mechanism by which OM-89 strengthens bladder epithelial antimicrobial defenses and enhances intracellular bacterial clearance, identifying enhanced lysosomal function as a therapeutically targetable component of host defense." in line 95.

In the discussion we changed: "Together, these findings provide a mechanistic framework for the long-observed clinical efficacy of OM-89. Our findings reveal that the urothelium itself can be

therapeutically targeted to reduce pathogen regrowth by transforming the epithelial barrier from a passive refuge for UPEC into an active defense site." to "Together, these findings provide a mechanistic framework for the long-observed clinical efficacy of OM-89 and identify epithelial lysosomal pathways as a therapeutically targetable component of host defense that can be used to improve intracellular bacterial clearance." in line 421 and "In the face of rising antimicrobial resistance (2024), strengthening epithelial antimicrobial function offers a complementary route to shift the bladder mucosa from a passive niche of bacterial survival and infection reactivation toward an active site of accelerated pathogen clearance." to "In the face of rising antimicrobial resistance (2024), our findings provide a mechanistic rationale for the clinical use of OM-89 and support epithelial lysosomal pathways as a promising target for host-directed therapeutic strategies that enhance intracellular bacterial clearance and improve the efficacy of existing antibiotics." in line 513.

2. Depending on the target audience, the Module-Module association analysis could need more introduction. I am not a computational biologist and it was not obviously apparent how Figure 4A is generated and what it actually showing. How specifically does this analysis demonstrate a functional link between lysosomal activity and immune defense pathways? Without further explanation, it is my opinion that this figure panel is an unnecessary distraction that is not required for any of the conclusions that the group can already draw from the rest of their data.

We thank the reviewer for this constructive critique. We agree that the rationale and interpretation of this analysis were not sufficiently explained in the original manuscript. We have therefore expanded the description of the MMAS approach and clarified how these data support the translational relevance of the lysosomal pathways identified in our experimental models.

Specifically, we now explain that the Module-Module Association Score (MMAS) analysis evaluates transcriptional correlations between the lysosomal gene network and functional biological pathways across eight independent human bladder transcriptomic datasets comprising more than 1,400 clinical samples. We further highlight the strong positive associations observed with host defense modules, including "response to molecule of bacterial origin", "cell activation involved in immune response", and "innate immune response". These additions clarify both the methodology and the rationale for including Figure 5A as a translational bridge between our experimental findings and human bladder biology.

The revised text (starting at line 251) now reads: "To test whether the observed effects on lysosomal pathways could mechanistically, at least in parts, explain OM-89-mediated protection, we first used Genebridge analysis (Li et al, 2019) to examine how the lysosomal gene signature identified in our RNA-seq data relates to host defense programs in the human bladder. To evaluate the translational relevance of our experimental findings, we used a computational Module-Module Association Score (MMAS) analysis across eight independent human bladder transcriptomic datasets comprising over 1,400 clinical samples. This network-based approach evaluates the transcriptional correlation between the lysosomal gene network and functional biological pathways across diverse human cohorts. Module-Module association analysis

performed on these human bladder datasets indicated that the lysosome module has strong positive associations with specific host defense modules, including "response to molecule of bacterial origin", "cell activation involved in immune response", and "innate immune response" (Figure 5A), highlighting a conserved functional link between lysosomal activity and immune defense pathways in the bladder epithelium. Altogether, these positive correlations suggest that enhanced lysosomal function represents a conserved pathway integrated within mucosal immunity across species, rather than an isolated cellular response unique to our experimental models."
