# Supplementary material for: Targeted lysosomal activation in bladder epithelium enhances clearance of intracellular uropathogenic Escherichia coli
Source: PLoS Pathog. 2026 Sep 25;22(9):e1014448. doi: 10.1371/journal.ppat.1014448 (PMC13614569; doi:10.1371/journal.ppat.1014448)
Supplement: Supplementary file 3 [file ppat.1014448.s017.pdf]

Response to Reviewers

Manuscript #PPATHOGENS-D-26-01662: Targeted lysosomal activation in bladder epithelium enhances clearance of intracellular uropathogenic *Escherichia coli*

Dear Dr. Subashchandrabose, Dr. Guillard, Dr. Bhaduri-McIntosh, and Dr. Malim,

Thank you for your letter and the opportunity to submit a revised version of our manuscript. Below we respond to the editor's comment. No reviewer comments were included with the decision letter and none were found as an attachment on Editorial Manager; we therefore have no reviewer points to address in this round.

Response to Additional Editor Comments

> *"Kindly add a working model schematic to summarize the key findings of this study as new Fig. 8."*

We thank the editor for this suggestion. We have added a new Fig 8, a working-model schematic that integrates the mechanistic findings of this study across both our mouse and human organoid/monolayer models. This new Fig 8 is referred to in the discussion. The figure and its legend are as follows:

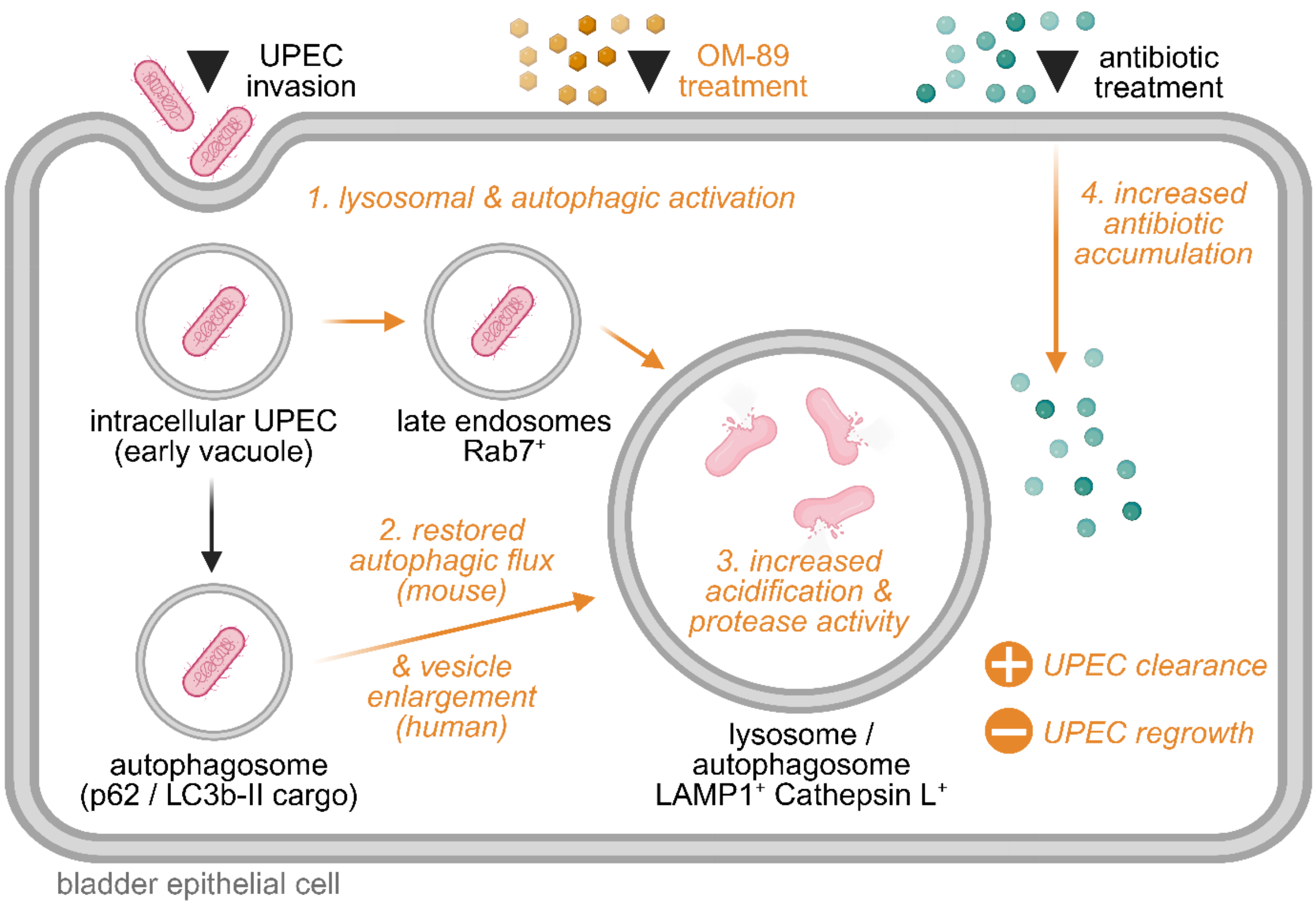

Fig 8. Working model hypothesis: OM-89 reinforces a lysosome-centered antimicrobial defense against intracellular UPEC in the bladder epithelium.

Schematic summary of the OM-89-induced antimicrobial response. OM-89 triggers coordinated activation of the endo-lysosomal and autophagic pathways in bladder epithelial cells. First, intracellular UPEC, internalized into early vacuoles or routed through p62/LC3B-II-positive autophagosomes, are trafficked through $Rab7^+$ late endosomes toward an expanded lysosomal compartment ($LAMP1^+$). Second, autophagic flux restoration is observed in mouse but not in human epithelial cells, but with enlarged lysosomal vesicles still observed in human cells. Third, OM-89 treatment increases lysosomal acidification and cathepsin L protease activity, enriching intracellular UPEC in proximity to proteolytically active autolysosomes. Fourth, in parallel, and independently of lysosomal routing, OM-89 increases intracellular accumulation of multiple antibiotic classes by an unknown mechanism. Together, these coordinated epithelial responses result in increased intracellular bacterial clearance and reduced post-antibiotic UPEC regrowth. Steps triggered by OM-89 are highlighted in orange. UPEC, magenta. Bladder epithelial cell, grey. Antibiotics, cyan. Created in BioRender. Tomasek, K. (2026) https://BioRender.com/wa2tl4q.

Response to Reviewers

No reviewer comments were provided with this decision.

We thank the editors again for their guidance and look forward to your assessment of our revised manuscript.

Sincerely,

Kathrin Tomasek, on behalf of all authors
